# It Is Not Always an Ectopic or Heterotopic Pregnancy

**DOI:** 10.7759/cureus.37083

**Published:** 2023-04-03

**Authors:** Ahmed Kamal Mohamed, Sakher Awadalla, Ali Nawaz

**Affiliations:** 1 Emergency Department, NMC Royal Hospital Khalifa City, Abu Dhabi, ARE; 2 Emergency Department, King’s College Hospital London, Dubai, ARE

**Keywords:** early pregnancy, uterus, rupture, hemoperitoneum, acute abdomen

## Abstract

Uterine rupture is a life-threatening peripartum complication. Spontaneous uterine rupture in early pregnancy is very rare. The diagnosis of uterine rupture should be considered when a pregnant patient presents with an acute abdomen because its clinical signs in early pregnancy are non-specific and the differentiation with other acute abdominal emergencies is challenging. Here, we present a case of acute abdominal pain. The patient was a 14-week pregnant 39-year-old female (gravida 4, para 2+1) with a history of two lower-segment cesarean sections. Our preoperative diagnosis was either heterotopic pregnancy or acute abdomen. Emergency laparotomy confirmed the presence of a spontaneous uterine rupture.

## Introduction

The rupture of a pregnant uterus is a life-threatening complication in obstetric practice with high maternal and fetal mortality. Uterine rupture (UR) can be complicated by hypovolemic shock, bladder injury, need for hysterectomy, and maternal death [1].

Early pregnancy UR is rare with several reported cases, but the actual rate of incidence is unclear in the current literature [2]. The risk of rupture in early pregnancy is increased in multipara with short pregnancy intervals [3] and in the presence of uterine scars [4]. Late diagnosis and a low index of suspicion can be life-threatening. In early pregnancy, most cases of hemoperitoneum are associated with an ectopic pregnancy. with UR being rarely suspected [5].

The classic symptoms described for third-trimester UR include acute-onset abdominal pain, vaginal bleeding, and non-reassuring fetal heart rate tracing. Unfortunately, these symptoms may not be present, especially with UR in early pregnancy [6].

Here, we describe the importance of including UR in the differential diagnosis of severe abdominal pain in early pregnancy by reporting a case of massive hemoperitoneum in a 14-week pregnant female due to spontaneous UR that was discovered during laparotomy.

## Case presentation

A 39-year-old pregnant female (gravida 4, para 2+1) with a gestational age of 14 weeks (based on her last menstrual period) presented to our emergency department with a history of acute, severe lower abdominal pain for 12 hours and vomiting three times. She had a history of two previous lower-segment cesarean sections (CSs), with the last one a year ago. There was no history of other uterine surgeries or procedures. She denied any history of trauma. She was on regular antenatal care at another healthcare facility.

**Figure 1:**
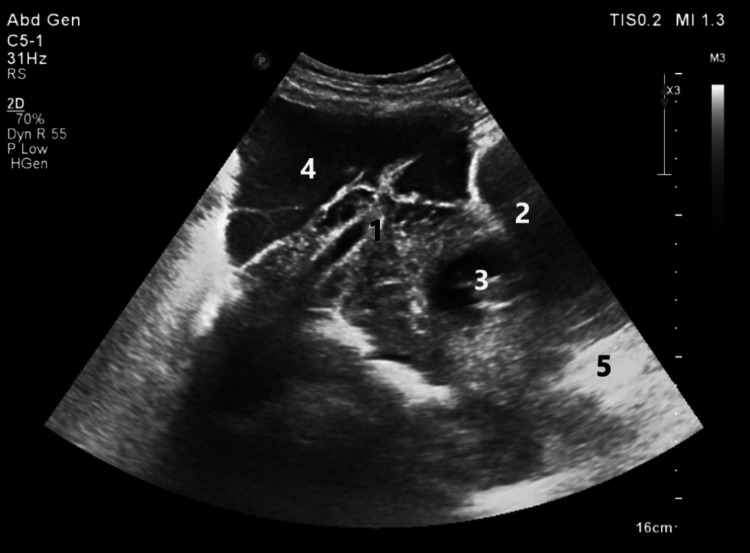
Bedside ultrasound image showing the rupture site with an ill-defined margin of myometrium and serosa of uterus fundus (1), urinary bladder (2), intrauterine viable fetus (3), pelvic free fluid with debris blood (4), and cervix (5).

On physical examination, the patient was conscious, oriented, and pale, and her limbs were cold and clammy. Her heart rate was 99 beats/minute, and her blood pressure was 100/70 mmHg. Abdominal examination revealed distension, guarding, and generalized tenderness with rebound tenderness all over the patient’s abdomen. The patient denied any vaginal bleeding, and per vagina examination was unremarkable. She was resuscitated, stabilized, and given analgesics. Urgent ultrasonography showed marked free fluid throughout the abdomen and pelvis (Figure 1).

**Figure 2:**
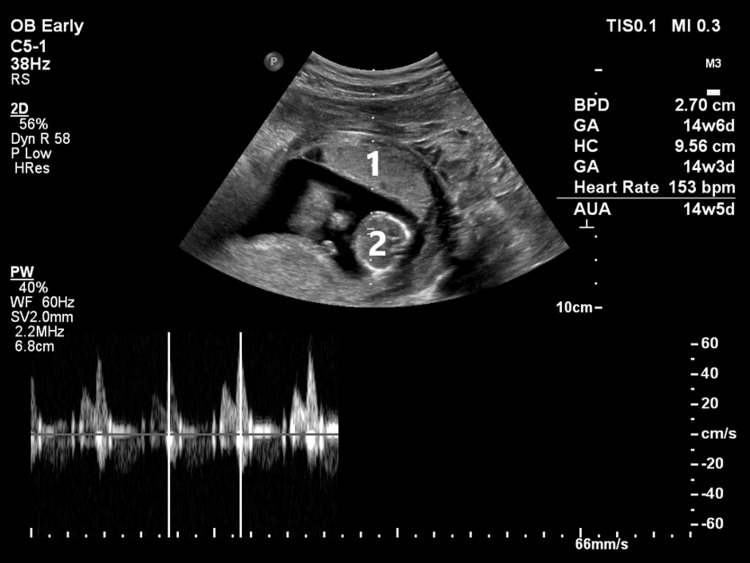
Bedside ultrasound image showing the anteriorly placed placenta with a small underlying hypoechoic area of retroplacental separation (1) and the cardiac area of an intrauterine viable fetus (2).

An intrauterine single viable fetus corresponding to 14 weeks and three days gestation and a hematoma were seen surrounding the bulky gravid uterus (Figure 2).

The ovaries were hardly differentiated from a sizable pelvic hematoma. As the patient started to show signs of shock, she was given blood and blood products. Laboratory investigations showed hemoglobin and platelet levels of 8.5 g/dL and 258 × 10^9^/L, respectively. The coagulation profile was normal. The patient was urgently referred to obstetrics and gynecology for the suspected rupture of a heterotopic pregnancy. The risk of abortion was explained, and signed consent was obtained. Exploration laparotomy was done under general anesthesia with the appropriate premeditations. It showed the complete rupture of the anterior wall of the uterus, extending from the fundus to the isthmus with a defect of approximately 6 cm. The fetus and placenta were found in the uterine cavity. However, a primary ultrasound scan showed an anteriorly placed placenta with a small underlying hypoechoic area of retroplacental separation. The defect was repaired with Vicryl-1 in three layers. The abdominal cavity was washed with normal saline. The ovaries and tubes were unremarkable, no tubal ligation was done, and the urinary bladder was intact. The estimated blood loss was nearly 1.8 L. Pregnancy was terminated, and the patient received three units of blood in the operation theater. There were no postoperative complications and the patient recovered well. She stayed for three days in the hospital and was charged with outpatient clinic follow-up. The four-week follow-up was satisfactory.

## Discussion

Spontaneous UR in early pregnancy is a rare but serious obstetric complication, especially if associated with a wrong or late diagnosis. Several cases have been reported in the literature [2,7-11]. The presence of a previous scar in the uterus is a very important risk factor for developing UR [12].

The early diagnosis of UR is difficult because typical clinical manifestations include abdominal pain, fetal heart irregularities, and vaginal bleeding [13]. These are non-specific findings and can occur in other conditions such as ectopic or heterotopic pregnancy [14]. A high level of suspicion must be maintained to avoid misdiagnosis of such a life-threatening complication. Our patient’s main complaint was severe abdominal pain. Most patients with UR in early pregnancy present with abdominal pain [2,11]. Though non-specific, patients may describe abdominal pain with a “ripping” sensation in the beginning. Palpating the abdomen to localize the area of maximal pain can aid in narrowing the diagnosis because UR causes midline pain [1]. Assessing vital signs, especially heart rate and blood pressure, is necessary because bleeding is usually intra-abdominal and not observed by the patient. For the same reason, the most important initial laboratory test is hemoglobin level.

Ultrasonography can be helpful. Finding an abnormality in the uterine wall, a hematoma next to a hysterotomy scar, free fluid in the peritoneum, anhydramnios, or fetal parts outside the uterus are findings supportive of UR diagnosis. However, the diagnosis is often confirmed when hemoperitoneum and fetal parts are identified outside the uterus during laparotomy [1]. Our patient had a completely ruptured uterus from the fundus to the isthmus. In the study by Perdue et al., the most common sites for UR were the fundus and the lower segment [2].

## Conclusions

UR should be considered in any pregnant woman with free fluid in the abdomen or hemoperitoneum, even when there are no identifiable risk factors in the obstetric history. Complete rupture of the uterus from the fundus to the isthmus can be encountered. Although UR in early pregnancy is rare, it must be kept in mind because early diagnosis and intervention can save the mother’s life.
